# The London Lancet

**Published:** 1868-07

**Authors:** 


### The London Lancet.

The undersigned take pleasure in announcing to the medical profession in the United States that, after considerable expense, they have arranged with the Proprietors for the publication of a special edition of the Lancet on thin paper, for circulation in America, thereby enabling them to make the above great reduction in price.

Subscriptions can commence with the new volume, which begins with the July number; copies of which will be sent regularly every week from this office.

Our edition of the Lancet besides containing nearly *one thousand* more pages than the re-print, (?) will include a number of original articles which no medical journal in this country can re-produce without violation of law.

We are maturing arrangements by which we will secure the contributions of original articles of the highest value to the profession. Such contributions will emanate only from the most distinguished Physicians and Surgeons in the United States. This plan, besides enhancing the excellence of the Lancet, already acknowledged as the ablest publication of its class in Great Britain, will operate as a legal barrier against the appropriation of the American articles by any publishers in the United States.

Taking into consideration the amount of valuable matter which we shall furnish our subscribers every week, the Lancet will be found not only the *best*, but the most *valuable* Medical Journal in the world.

Terms of Subscription.—One copy, postage pre-paid, per annum, $12.00 currency. One copy, postage pre-paid, six months, $6.00 currency, in advance. Specimen copies forwarded on application to

KELLY & PIET,
No. 174 West Baltimore street, Baltimore,
Agents for the United States.

